# Clinicopathological analysis of oral and perioral keratoacanthoma: a systematic review based on case reports and case series

**DOI:** 10.4317/medoral.27631

**Published:** 2026-01

**Authors:** Carolina Louzada Menna-Barreto, Laura Borges Kirschnick, Lauren Frenzel Schuch, Fabio Muradás Girardi, Márcio Ajudarte Lopes, Manoela Domingues Martins

**Affiliations:** 1 Department of Oral Pathology, School of Dentistry, Federal University of Rio Grande do Sul, Porto Alegre, RS, Brazil; 2 PhD, Oral Diagnosis Department, Piracicaba Dental School, University of Campinas, Piracicaba, SP, Brazil; 3 PhD, Department of Diagnosis in Pathology and Oral Medicine, School of Dentistry, Universidad de la República (UDELAR), Montevideo, Uruguay; 4 DDM, Department of Oral Pathology, School of Dentistry, Federal University of Rio Grande do Sul, Porto Alegre, RS, Brazil; 5 PhD, Oral Diagnosis Department, Piracicaba Dental School, University of Campinas, Piracicaba, SP, Brazil; 6 PhD, Department of Oral Pathology, School of Dentistry, Federal University of Rio Grande do Sul, Porto Alegre, RS, Brazil

## Abstract

**Background:**

To systematically review the literature on clinicopathological features of keratoacanthomas (KA) in the oral and perioral region.

**Material and Methods:**

The review protocol as registered under the number CRD42022323274. Electronic searches were conducted in four databases with a specific search strategy using MeSH and free terms.

**Results:**

60 studies reporting 73 cases of KA were included in this systematic review. KA affects more men with a mean age of 49.09 years old. Sun-exposed area of the lower lip was the most common anatomical location of the perioral lesions (38.35%) and the oral mucosa (9.58%) of the intra-oral ones. The most frequent clinical presentation of the perioral lesions was of an ulcerated nodule (36.23) and intra-orally of an papule (11.59) or nodule (11.59). Incisional biopsies were the commonest procedure for the diagnosis. The histological findings commonly presented a hyperplastic epithelial tissue forming a crater-like structure filled with keratin. Slightly atypia, keratin pearls and an inflammatory infiltrate in connective tissue could also be seen. Surgical excision was the treatment of choice in the majority of cases (45.71%). A low recurrence rate (6.89%) was seen in a mean follow-up time of 20.54 months.

**Conclusions:**

KA affects commonly sun-exposed areas of men in their sixth decade of life. Given the similarity of squamous cell carcinoma, it is important to study KA to help clinicians with the correct diagnosis and treatment.

## Introduction

The term keratoacanthoma (KA) was first reported by Dr. Hutchinson in 1889. KA is usually described as a rapid growing nodule with a central crater. This lesion is benign and has the potential to involute spontaneously (1 , 2). The etiology of KA is still unknown. There has been discussion regarding the association to many factors as trauma, sunlight exposure or immunosuppressive drugs. Dr. Svirsky (1977) considers the possibility of their origin on the sebaceous glands, especially when KA occurs on the buccal mucosa. Moreover, some authors defend the origin from hair follicles, although this cannot justify the development of lesions in the oral cavity (1 , 3 - 5).

KA frequently affects men between the ages of 50 and 69 years, and it is commonly located in sun-exposed areas of skin, such as the face, neck and hands (6 , 7). The main anatomical location of solitary intraoral KAs is the anterior maxilla (8). In regard to its multiple form, the buccal mucosa was the preferred location of KA development (9 - 14). KA lesions have three clinical stages: proliferative, mature and resolving. The clinical presentation of both cutaneous and intra-oral KA during the diagnostic process is mainly of a nodule which indicates that the lesions were diagnosed mostly in the proliferative and mature stages (4 , 6 , 15).

Histopathologically, KA shows a central crater filled with keratin, irregular epidermal proliferations, cells with keratinization or pearl formation, and a dense mixed infiltrate. In rare cases, it can also exhibit mitosis and atypical cells (5 , 7 , 16). Furthermore, KA has several histological similarities with squamous cell carcinoma (SCC) -the most common cancer of the oral cavity and lips-, which caught attention due to the distinct behavior of each lesion (17 - 19).

Although KA rarely affects the oral cavity, there are case reports and case series of its occurrence published in the literature. Given the significant differential diagnosis with SCC and its rarity in the oral cavity, the aim of this study is to compile information on cases of oral and perioral KA and discuss the clinicopathological characteristics and treatment options to aid clinicians when faced with a suspicion of KA.

## Material and Methods

Eligibility criteria

The inclusion criteria of this systematic review were based on Patient-Exposition-Comparators-Outcomes-Study design (PECOS) acronym: (P) patients with KA in the oral and perioral region; (E) KA; (C) not applicable; (O) define the main clinicopathological features of oral or perioral KAs (S) case report and case series. The study selection was based on case reports or case series that presented a clear description of the morphological findings and/or histopathological photomicrographs confirming the diagnosis. Conference abstracts, unavailable studies, lesions in other regions than oral and perioral location, duplicated samples and studies in languages other than English were excluded. Letters to the editor -only if containing all patient's information- were included in the systematic review.

Information sources and search strategy

The electronic searches had no restrictions on year of publication and were performed in June 2023. Individual search strategies were designed for the following electronic databases: MEDLINE/PubMed, EMBASE, Web of Science and Scopus and the search strategy, containing a combination of predefined Medical Subject Heading (MeSH) terms and free terms (Supplement 1. http://www.medicina.oral.com/carpeta/suppl1_27631). Gray literature was searched on Google Scholar. Additionally, a manual search of the reference lists of the included studies to locate any unidentified study during the conventional search was performed.

Selection, data and collection process

All of the studies were reviewed by two authors (C.L.M.B. and L.B.K.). If they met the eligibility criteria, the study was included. Possible disagreements between the two authors were solved consulting a third one (L.F.S.). The following information was extracted of the selected studies: Publication details (first author, year of publication and country); patients' gender; patients' age; patients' habits; location of the lesion; clinical characteristics; type of biopsy; histopathological features immunohistochemical features; treatment; follow-up time and recurrence.

Study risk of bias assessment

Critical appraisal of the included articles was made using the Joanna Briggs Institute - University of Adelaide tool for case reports or case series (20). The evaluation of the studies was made independently by two reviewers (C.L.M.B. and L.B.K). Disagreements were resolved consulting a third author for arbitration (L.F.S.).

Other information

This systematic review was carried out according to the Preferred Reporting Items for Systematic Reviews and Meta-analyses (PRISMA) guidelines (21). A protocol was drafted and registered in the National Institute for Health Research's International Prospective Register of Systematic Reviews (PROSPERO) under the number: CRD42022323274.

## Results

Study selection

The electronic searches in the databases totaled 618 references. After the exclusion of 266 duplicates, titles and abstracts of 352 studies were evaluated. Inclusion and exclusion criteria were applied, and 146 full-text articles were read. Of these, 100 articles were excluded for the reasons described in Supplement 2 (http://www.medicina.oral.com/carpeta/suppl2_27631).

**Figure 1:**
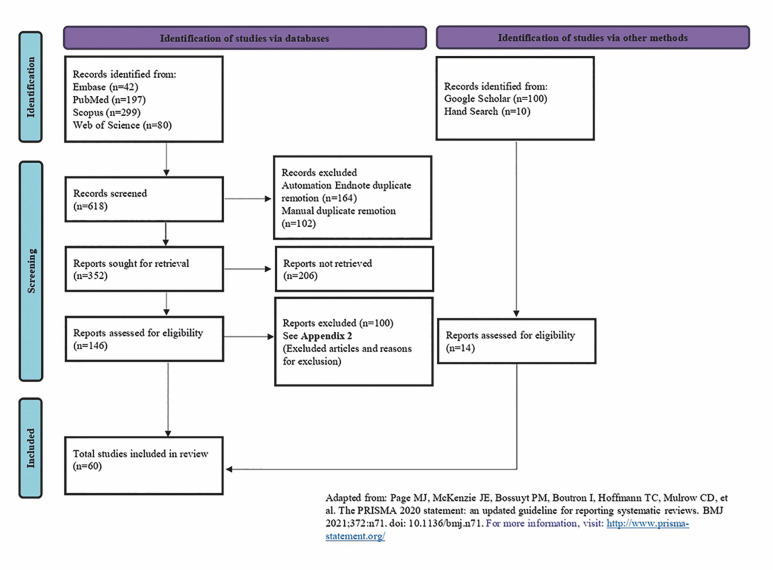
PRISMA flow diagram for systematic search and study selection strategy.

A hand search was conducted by cross-checking the references of all included studies, and 10 new articles were added in this systematic review. Gray literature reached 4 more articles. In the end of the selection process, 60 articles reporting 73 cases of oral and perioral KA were included in this systematic review (references on Supplement 3. http://www.medicina.oral.com/carpeta/suppl3_27631). Figure 1 depicts the search and selection process

1

General characteristics of the included studies

Of the 60 included articles, 47 were case reports and 13 were case series - reporting more than 1 case in the same article. The studies were published between 1958 and 2022 in 20 different countries, as follows: United States (n=16), China (n=7), Brazil (n=5), India (n=6), Japan (n=3), Australia (n=2), Canada (n=3), Germany (n=3). Netherlands, Italy, Israel, Jamaica had two studies each and, United Kingdom, Arabic Emirates, Croatia, Egypt, Iran, Korea, New Zealand and Puerto Rico had one study published in each country.

Demographics

Table 1 summarizes the general characteristics of the cases included in this systematic review. Males (n=49/69.01%) were more affected with a mean age of 49.09±17.97 years old.

Table 1

Anatomic location, clinical features and biopsy procedure

**Figure 2:**
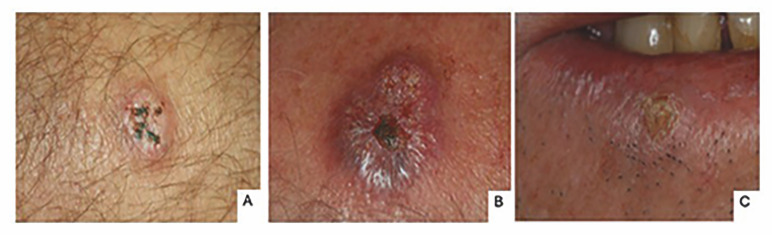
Clinical aspects of KA in the skin and perioral region. Solitary nodule with ulceration and crust in skin (A and B) and lower lip (C).

The most affected location in the perioral region was the lower lip skin (n=28/38.35%), followed by upper lip skin (n=18/24.65%) with a clinical presentation of an ulcerated nodule (n=25/36.23%) (Figure 2). In the oral cavity, the main anatomical location was the oral mucosa not otherwise specified (n=7/9.58%) followed by gingiva (n=6/8.21%) and buccal mucosa (n=5/6.84%) presenting as papules (n=8/11.59) or nodules (n=8/11.59). Regarding symptoms, symptomatic lesions were most common (n=20/64.51). Biopsy was performed in 46 cases and an incisional biopsy was most frequently done (n=23/50.00%).

2

Histopathological and immunohistochemical features

**Figure 3:**
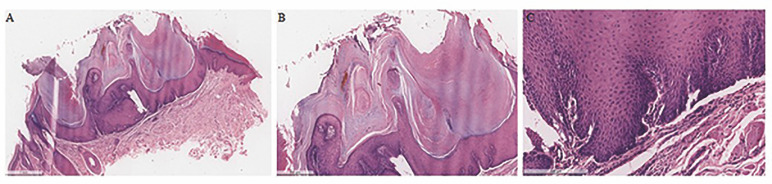
Histological Features of Keratoacanthoma. (A) Low-power magnification (H&amp;E, 40×) reveals the characteristic morphological features of keratoacanthoma, including well-defined hyperplastic, crateriform endophytic epithelium. (B) The tumor nests are sharply demarcated from the surrounding stroma, with tumor cells displaying prominent nucleoli and eosinophilic, glassy cytoplasm. (C) A higher magnification (H&amp;E, 200×) highlights a dense chronic inflammatory infiltrate (arrows) within the connective tissue adjacent to the tumoral epithelium. Additionally, actinic elastosis is observed near the lesion, along with nuclear hyperchromatism (outlined), which is a typical feature of keratoacanthomas.

All cases presented detailed microscopic aspects or histopathological images. KA often exhibits as an exophytic surface with hyperplastic squamous epithelium with or without a keratin layer, forming a central crater-like structure filled with keratin. Features such as dyskeratosis, mild atypia or pleomorphism, acanthosis and a chronic inflammatory infiltrate in the connective tissue were also observed. Sometimes epithelial islands containing keratin pearls or cells with a "glassy" cytoplasm could be seen. (n=12) (Figure 3).

3

Information about immunohistochemistry was present only in four cases and in all of them was used to exclude the diagnostic hypothesis of SCC. For these, epithelial markers, such as cytokeratin (CK), were used in three cases, and Ki-67 index marker was used in one case.

Treatment, recurrence and follow-up

Surgical excision was the commonest treatment choice (n=32/45.71%) and the majority of the cases had no history of recurrence (n=54/93.10%) The mean follow-up time was 20.54±26.26 months, ranging from 2 weeks to 120 months.

Study risk of bias assessment

Concerning the case reports, patients' demographic characteristics were clearly described in all studies. One study did not clearly describe patients' history as a timeline and their current clinical condition. Diagnostic tests and their results, intervention and post-intervention clinical conditions were not clearly described in some studies. Adverse and unanticipated events were not identified in 90% of the studies, and only one study did not provide any takeaway lessons. Regarding the case series, all of them described a clear inclusion criterion, the demographic information of the patients, a standardized, reliable measurement of the patients' conditions, valid methods for identification of the condition, as well as consecutive and complete inclusion of participants. Only 7 studies did not clearly report clinical information of the participants. The outcomes and the follow-up results of the cases and the presenting site(s)/clinic(s) demographic information of cases were clearly reported in all of these studies. Statistical analysis was not applicable for all studies (Supplement 4 http://www.medicina.oral.com/carpeta/suppl4_27631).

## Discussion

KA is a benign lesion commonly found in sun-exposed areas; however, it can also affect the oral and perioral regions (3 , 4). While KA is rare in the oral cavity, clinicians must be familiar with the clinical and histopathological characteristics, as well as treatment, due to its very similar histological appearance to well-differentiated SCC (17 , 22 - 24). To the best of our knowledge, this is the first systematic review of KA in the oral and perioral region, bringing together 73 cases to assist clinicians in the diagnostic process.

Our study revealed that men were more affected by KA, with a mean age of 49.09 years. This fact corroborates with the slight prevalence of KA in male individuals, particularly between their sixth and eighth decades of life, as observed in previous studies (6 , 15 , 17). Among the common habits identified in this systematic review, smoking, alcohol consumption, and sunlight exposure were the most prevalent. Although the etiology of KA remains unclear, previous studies suggest a potential link to sun radiation, highlighting ultraviolet radiation as a significant factor. Additionally, Karaa and colleagues (2007) discuss the involvement of other risk factors such as exposure to cigarette carcinogens and trauma and the development of KA lesions (5 , 6 , 15 , 17).

In our study, the most affected anatomical location was the lower lip skin, a sun-exposed area frequently impacted by KA (6 , 17 , 23 , 25). Intraorally, when solitary, the most common site was the gingiva, in contrast to the found in a study published in the literature, where the anterior maxilla is often reported (8). When multiple, the oral mucosa was the most affected site. This fact was consistent with the literature, which presents that, in multiple form, KA can affect many mucous membranes, such as oral. Furthermore, this multiple form usually involves other parts of the body and are commonly associated with genetic disorders (3 , 10 , 17). However, due to the rarity of oral involvement and the limited number of studies on intraoral cases, this information may carry some bias.

The clinical presentation of the cases in this systematic review was predominantly of a nodule, particularly in the solitary form of KA, aligning with previous studies (6 , 7 , 22). Literature categorizes the development of the lesion into three stages - proliferative, mature, and resolving. The proliferative and mature forms are more commonly diagnosed, and they present as a nodule (4 , 6 , 15). Additionally, over 50% of the cases (n=20) were symptomatic. Although some studies described KA as a painless nodule, others reported symptoms such as pruritus or pain (22 , 26 , 27).

Histopathologically, KA exhibits a surface with hyperplastic squamous epithelium with a central crater-like structure filled with keratin, as demonstrated in our study. Additionally, features such as cell keratinization, mild atypia or pleomorphism, acanthosis, keratin pearls, and a chronic inflammatory infiltrate in the connective tissue were observed. These findings align with numerous studies in the literature that describe similar histological characteristics of KA (5 , 7 , 22 , 26).

No significant immunohistochemical findings were reported in the five studies of this systematic review that used this technique (28 - 32). The markers were used mainly to compare results between KA and SCC and to analyze the potential of tumor-cell invasion. Ki-67 can contribute to the investigation of the tumor clinical stage and aggressiveness since studies showed that this index has a higher expression in malignant lesions when compared to benign lesions or normal tissues. (33 , 34). Another example is the CK staining, which is an epithelial marker and can be used to evaluate the cells differentiation aspect and is important to indicate malignancy (35). Thus, KA does not have a specific marker and a meticulous histopathological analysis associated with the patient follow-up are recommended (4 , 17 , 18).

The surgical excision was the predominant treatment in most cases (n=32). The literature presents a dilemma concerning the treatment of KA, with some suggesting clinical follow-up as a viable option due to its potential for spontaneous involution (5 , 36). In our survey, all 7 cases that opted for clinical follow-up exhibited spontaneous involution within an average time of 19.4 months. While surgical excision, coupled with a thorough histological analysis, holds significance due to the resemblance of KA to SCC, certain considerations should be addressed (4). Firstly, it is crucial to emphasize the importance of an incisional biopsy when faced with suspicions of SCC, particularly for clinicians, such as dentists (37 , 38). Secondly, the skin areas of the lips hold aesthetic and functional relevance, influencing patient adherence to treatment and the necessity of surgical intervention (39). Therefore, we recommend for meticulous analysis of an incisional biopsy specimen before determining the course of treatment.

The majority of cases showed no signs of recurrence during the follow-up period. Existing literature supports the fact that KA has a low rate of recurrence, particularly after surgical excision (40). In our study, out of the cases that underwent surgical excision, only two cases experienced recurrence. The follow-up duration for the patients (n=49) ranged from 2 weeks to 120 months, with a mean duration of 20.54 months. This mean follow-up time aligns with the findings of Dr. Aubut and colleagues (2012), who conducted a retrospective study involving 46 cases of KA lesions in general locations (24).

This systematic review has a noteworthy limitation to address. The incompleteness of patients' data in the reports may have impacted our results. The review's dependency on how authors described each case led to varying levels of information clarity. For instance, in case of symptoms, only 31 out of the 73 cases provided this information, highlighting a potential confounding factor due to the amount of unreported data.

## Conclusions

Our analysis showed that KA rarely affects the oral cavity. Its main anatomical location in the perioral region is the lower lip skin, and inside the oral cavity is the oral mucosa, both of middle-aged men. While intraoral KA tends to be papular or nodular in form, perioral KA usually presents as ulcerated nodules. Although KA has a tendency to spontaneous regression, surgical excision was the most common treatment. The lesions showed a low potential of recurrence during follow-up time (20.54 months). It is important to correctly diagnose KA lesions due to their clinicopathological similarity with subtypes of SCC, to choose the correct treatment for the patients.

## Figures and Tables

**Table 1:** Table Summarized features about the cases of keratoacanthoma included in the systematic review.

Variables			n (%)
Sex (n=71)		Male	49 (69.01)
		Female	22 (30.98)
Age (±SD) (n=73)			49.09±17.97Range: 7 to 81 years old
Habits (n=21)*		Smoke	14 (66.66)
		Sun-expose (outside worker/farmer/driver)	11 (52.38)
		Alcohol consumption	5 (23.80)
Anatomical locations (n=73)**	Perioral (n=59)	Lower lip - Skin	28 (38.35)
		Upper lip - Skin	18 (24.65)
		Above the upper lip	5 (6.84)
		Chin/below the lower lip	3 (4.10)
		Lips not otherwise specified	2 (2.73)
		Commissure	2 (2.73)
		Alveolus	1 (1.36)
	Intra-oral (n=26)	Oral mucosa not otherwise specified	7 (9.58)
		Gingiva	6 (8.21)
		Buccal mucosa	5 (6.84)
		Tongue	4 (5.47)
		Palate	3 (4.10)
		Labial mucosa	1 (1.36)
Lesions (n=73)		Solitary	59 (80.82)
		Multiple (not exclusively in the oral or perioral region)	14 (19.17)
Symptoms (n=31)		Yes	20 (64.51)
		No	11 (35.48)
Biopsy (n=46)		Incisional biopsy	23 (50.00)
		Excisional biopsy	12 (26.08)
		Biopsy not otherwise specified	11 (23.91)
Clinical presentation (n=69)***	Perioral (n=49)	Ulcerated nodule	25 (36.23)
		Nodule	10 (14.49)
		Tumor	6 (8.69)
		Ulcer	6 (8.69)
		Papule	1 (1.44)
		Erosion	1 (1.44)
	Intra-oral (n=25)	Papule	8 (11.59)
		Nodule	8 (11.59)
		Ulcerated nodule	5 (7.24)
		Ulcer	3 (4.34)
		Plaque	1 (1.44)
Treatment (n=70)****		Surgical excision	32 (45.71)
		Excisional biopsy as treatment	11 (15.71)
		Retinoid	8 (11.42)
		No treatment	7 (10.00)
		Chemotherapy	6 (8.57)
		Curettage	3 (4.28)
		Photodynamic therapy	3 (4.28)
		Radiotherapy	2 (2.85)
		Vitamins	2 (2.85)
		Corticoid	2 (2.85)
		Antihistamine	2 (2.85)
		Other drugs	2 (2.85)
		Antiviral	1 (1.42)
Recurrence (n=58)		No recurrence	54 (93.10)
		Recurrence	4 (6.89)
Follow-up (n=49)			20.54±26.26Range: 2 weeks to 120 months

*A patient may have more than one habit. **Some patients presented more than one lesion. *** Some patients presented more than one lesion and, consequently, more than one clinical presentation. ****Not all cases described the lesion’s treatment. One case may have had more than one treatment choice.

## Data Availability

Declared none.
